# *iso*-Petromyroxols: Novel Dihydroxylated Tetrahydrofuran Enantiomers from Sea Lamprey (*Petromyzon marinus*)

**DOI:** 10.3390/molecules20035215

**Published:** 2015-03-23

**Authors:** Ke Li, Cory O. Brant, Ugo Bussy, Harshita Pinnamaneni, Hinal Patel, Thomas R. Hoye, Weiming Li

**Affiliations:** 1 Department of Fisheries and Wildlife, Michigan State University, East Lansing, MI 48824, USA; E-Mails: like4@msu.edu (K.L.); brant@msu.edu (C.O.B.); bussy@msu.edu (U.B.); pinnama3@msu.edu (H.P.); patelhi3@msu.edu (H.P.); 2 Department of Chemistry, University of Minnesota, 207 Pleasant Street SE, Minneapolis, MN 55455, USA; E-Mail: hoye@umn.edu

**Keywords:** stereoisomers, relative configuration, cyclostomata

## Abstract

An enantiomeric pair of new fatty acid-derived hydroxylated tetrahydrofurans, here named *iso*-petromyroxols, were isolated from sea lamprey larvae-conditioned water. The relative configuration of *iso*-petromyroxol was elucidated with 1D and 2D NMR spectroscopic analyses. The ratio of enantiomers (*er*) in the natural sample was measured by chiral-HPLC-MS/MS to be *ca.* 3:1 of (–)- to (+)-antipodes.

## 1. Introduction

**Figure 1:**
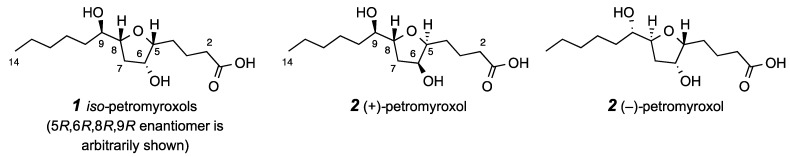
Structures of *iso*-petromyroxols (**1**, this work) and petromyroxols (**2**, [18]).

Sea lamprey (*Petromyzon marinus*) is a useful model for a wide range of research topics, such as chemical communication [1,2], behavior [3,4], neurobiology [5,6,7], ecology [8], and evolution [9,10]. Its metabolite profile is known to shift through its life stages [11,12]. The structural diversity of known sea lamprey metabolites is particularly complex and interesting [4,13,14,15,16,17]. Here we report the relative configuration of two enantiomeric secondary metabolites, which we have named *iso*-petromyroxol (**1**) (Figure 1).

In a previous investigation [18], we have described the isolation, structure determination, and preliminary biological activity (olfactory response) of (+)- and (–)-petromyroxols (**2**). These compounds were isolated from larval sea lamprey-conditioned water. The central tetrahydrofuran (THF) ring in **2** bears a *trans*-relationship between the two carbon substituents bound to C-5 and C-8. Compounds containing hydroxylated THF subunits have been found in terrestrial plants [19], marine alga [20,21,22], and marine mollusks [23]. Petromyroxols (**2**) represent the first examples of dihydroxylated THF-containing metabolites isolated from a vertebrate [18]. Through further studies we have deduced the structures of a related and more minor pair of metabolites, here named *iso*-petromyroxol (**1**), which is a diastereomer of **2**.

## 2. Results and Discussion

Compound **1** was isolated as a colorless oil. High resolution mass spectrometry (HR-MS) signals were observed at 273.1702 Da [(M−H)^−^ calculated for C_14_H_25_O_5_, 273.1707 Da] and 297.1678 Da [(M+Na)^+^ calculated for C_14_H_26_O_5_Na, 297.1672 Da] for the negative and positive modes, respectively, of the electrospray ionization spectrum (ESI^+/−^). The ∆*m/z* of 0.5 mDa (1.8 ppm) and 0.6 mDa (2.0 ppm) for [M−H]^−^ and [M+Na]^+^ suggested C_14_H_26_O_5_ as the parent formula of compound **1**.

**Figure 2:**
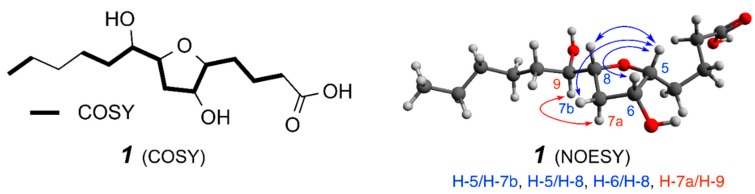
Key COSY and NOESY correlations for *iso*-petromyroxols (**1**).

^1^H and ^13^C-NMR data, in particular the similarity in chemical shifts (Table 1), suggested that compound **1** possessed a structure related to that of the petromyroxols **2** [18]. ^1^H-^1^H COSY analysis indicated that compounds **1** and **2** shared the same connectivity throughout the central C-2 to C-10 region of the molecules, indicating that **1** and **2** are diastereomers. A detailed analysis of the NOESY spectrum of **1** revealed correlations between H-5/H-7b, H-5/H-8, and H-6/H-8 (Figure 2), placing all four of these protons on the same face of the tetrahydrofuran ring. A complementary NOESY correlation was observed between H-9/H-7a, indicating their *cis* relationship to one another.

**Table 1:** NMR spectroscopic data (^1^H NMR, 600 MHz and ^13^C-NMR, 225 MHz in CDCl_3_) for *iso*-petromyroxols (**1**) and petromyroxols [18] (**2**).

No.	*iso-*petromyroxols (1)		petromyroxols (2)	
	δ_C_	δ_H_, mult (*J* in Hz)	δ_C_	δ_H_, mult (*J* in Hz)
1	176.5	-	177.6	-
2	33.5	2.44 (t, 6.7)	33.7	2.43 m (Σ*J*s = 18)
3	21.4	1.73 m	21.4	1.77 m, 1.70 m
4	28.0	1.72 m	28.4	1.72 m, 1.67 m
5	83.8	3.66 (ddd, 2.8, 6.2, 6.2)	82.5	3.79 ddd (*ca.* 2.5, 6.5, 6.5)
6	71.6	4.10 (dd, 5.2, 2.7)	73.5	4.30 dd (*ca.* 3.5, 3.5)
7a	38.4	1.85 (dd 14.2, 3.5)	37.8	1.89 ddd (4.6, 9.2, 13.7)
7b		2.39 (ddd, 5.6, 9.9, 14.0)		2.02 dd (6.6, 13.4)
8	79.3	3.98 (ddd, 2.2, 3.8, 10.0)	80.7	4.06 ddd (6.5, 6.5, 8.9)
9	73.9	3.49 (ddd, 2.4, 3.9, 8.2)	74.3	3.39 m (Σ*J*s = 18)
10	34.2	1.52 m	33.3	1.40 m
11	25.7	1.44 m, 1.35 m	25.4	1.51 m, 1.38 m
12	31.7	1.28 m	32.0	1.29 m
13	22.6	1.30 m	22.8	1.31 m
14	14.0	0.89 t (7.0)	14.2	0.89 t (6.9)

**Figure 3:**
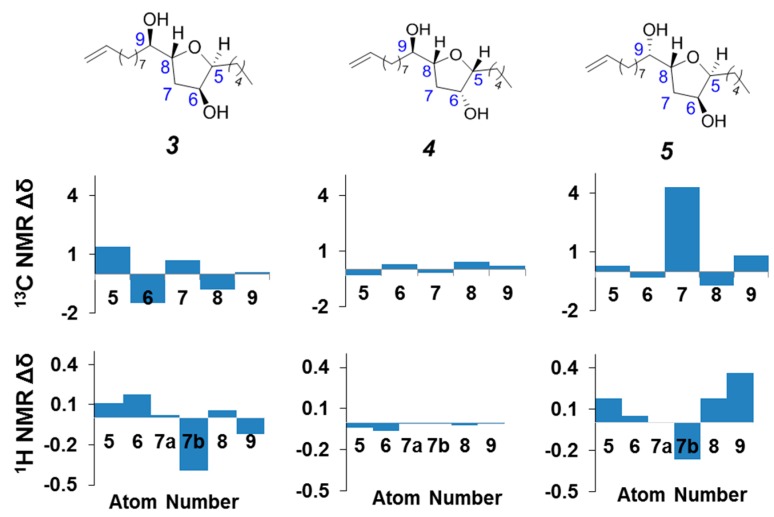
Differences in carbon (upper set of graphs) and proton (lower set of graphs) chemical shifts between *iso*-petromyroxols (**1**) and the analogs **3**, **4**, and **5**, respectively, at positions C-5 through C-9 (*iso*-petromyroxol skeleton numbering used for clarity).

The closest known structural analogues to compound **1** are the dihydroxylated THFs **3**–**5**. The first two are natural products, isolated from a brown algae found in Australia [24,25], and the third is a synthetic analog **5** [26]. Differences in the carbon and proton chemical shifts between **1** (Figure 2) and each of **3**–**5** are shown graphically [27,28,29] in Figure 3. These comparisons clearly suggest that the best match is between **1** and **4**, as **4** also possesses an 5R*,6R*,8R*,9R* relative configuration [25]. These results further validated our assignment of the structure of *iso-*petromyroxols.

**Figure 4:**
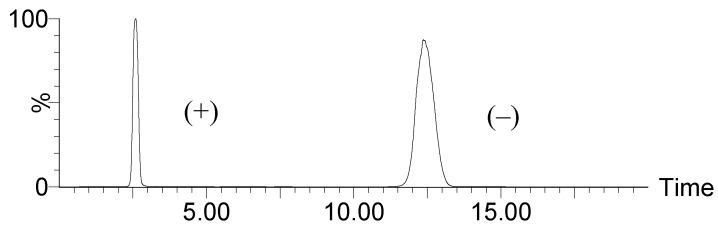
Analysis of (+)- and (–)-*iso*-petromyroxols by chiral HPLC-MS/MS on APCI positive mode.

As was the case for the petromyroxols (**2**) [18], the natural sample of **1** showed a non-zero specific optical rotation (${\left[\text{α}\right]}_{D}^{25}$ −5.0, *c* 0.10, CH_3_Cl), indicating that the sample of **1** was non-racemic. Analysis by chiral HPLC (Diacel Chiralpak^®^ AD-H, tandem atmospheric-pressure chemical ionization mass spectrometry (APCI-MS/MS detection)) showed that the sample comprised a substantial amount of both enantiomers of **1** (Figure 4). The material was separated to provide *ca.* 300 µg of each antipode. The earlier-eluting enantiomer showed a specific rotation of ${\left[\text{α}\right]}_{D}^{25}$ = +10 (*c* 0.20, CHCl_3_), and the latter ${\left[\text{α}\right]}_{D}^{25}$ = −12 (*c* 0.20, CHCl_3_). The enantiomeric ratio (*er*) was *ca.* 25% of (+)-**1** to 75% (−)-**1** as deduced from peak integration of the chromatogram.

## 3. Experimental Section

### 3.1. General

1D and 2D NMR spectra of **1** were recorded on a Bruker 900 MHz or Agilent 600 MHz spectrometer. Mass spectra were measured on a TQ-S TOF LC mass spectrometer (Waters Corporation, Milford, MA, USA). Optical rotation values were determined on a polarimeter model 341 (Perkin Elmer, Waltham, MA, USA) Chromatography supports—silica gel (70–230 and 230–400 mesh), RP-18 reverse-phase silica gel, and Sephadex LH-20—were obtained from E. Merck (Darmstadt, Germany). TLC was performed on glass plates pre-coated with GF254 silica gel, also obtained from E. Merck. Analytes were first visualized with 254 nm light and then stained with a spray of 5% anisaldehyde (Sigma-Aldrich, St. Louis, MI, USA) in acidic methanol.

### 3.2. Animals

Procedures involving sea lamprey larvae were conducted in accordance with the Public Health Service (PHS) Policy on Humane Care and Use of Laboratory Animals, incorporated into the Institute for Laboratory Animal Research Guide for Care and Use of Laboratory Animals, and have been approved by the Institutional Animal Use and Care Committee at Michigan State University (Animal use form number: 03/11-053-00). Larval sea lampreys were collected from tributaries of the Laurentian Great Lakes by the Fisheries and Oceans Canada and the US Fish and Wildlife Service according to approved collection permits from the respective government agencies. The larvae were transported to the USA Geological Survey Hammond Bay Biological Station in Millersburg, Michigan, state abbre, USA. The animals were held in flow through tanks (0.6 m deep by 1.8 m wide by 15.2 m long).

### 3.3. Extraction of Larval Sea Lamprey Conditioned Water

Every 4–5 days over the course of *ca.* 5 months, sea lamprey larvae-conditioned water (*ca.* 4000 L for each cycle), from the tank holding *ca.* 8000–10,000 larvae was passed through four column beds each containing 1 kg of Amberlite XAD 7HP resin. The flow rate was *ca.* 200 mL/min/bed. Each column was subsequently eluted with 4 L of methanol at each cycle. The eluent was processed at 40 °C under reduced pressure using a rotary evaporator, yielding *ca.* 1 L solution that comprised primarily water. These solutions were pooled and stored at −80 °C until further processing. A 42 L pool of these combined solutions was thawed and concentrated by lyophilization. The resulting residue was suspended in methanol and filtered through 2 µm filter paper to remove the insoluble portion. The filtrate was concentrated again under reduced pressure at 40 °C, yielding 2.0 g of a dark residue.

### 3.4. Purification of iso-Petromyroxol (**1**)

The above dark residue was subjected to preparative chromatography over silica gel (150 g) using a gradient of chloroform:methanol, stepped from 10% to 100% methanol. Thin layer chromatography (TLC) analysis was used to pool the eluants into nine fractions. Fraction 4, after concentration, yielded *ca.* 26 mg of a brown oil. This was further purified using two successive Sephadex LH-20 columns that were eluted with CHCl_3_-MeOH (1:1) and MeOH (100%), respectively. The sample of the non-racemic mixture of *iso*-petromyroxols (**1**, 1.4 mg) was identified and its purity enrichment judged and guided by TLC.

*iso*-petromyroxol (**1**): colorless oil; ${\left[\text{α}\right]}_{D}^{25}$ −5.0 (*c* 0.10, MeOH); ^1^H and ^13^C-NMR data, see Table 1; ESI-MS *m/z* (%) 273 (3), 193 (5), 168 (5), 145 (7), 143 (14), 129 (100), 127 (25), and 125 (12); HR-ESI-MS *m/z* 273.1702 (calcd for C_14_H_25_O_5_, 273.1709 [M−H]^−^).

### 3.5. Chiral HPLC-MS/MS Analysis

A HPLC-MS/MS method was developed to achieve baseline separation of the enantiomers of 1. These analyses were carried out on a Waters ultra-performance liquid chromatography (ACQUITY UPLC^®^) system fitted with a Xevo TQ-S tandem quadruple mass spectrometer (Waters) that used an APCI source in the positive mode. The mixture of 1 was analyzed on a Chiralpak^®^ AD-H column (2.1 × 150 mm, 5 μm). Isocratic elution with *n*-hexane:ethanol:formic acid (85:15:0.1, v/v/v) was performed at a flow rate of 0.70 mL/min. Multiple reaction monitoring (MRM, 275.0 Da parent and 238.9 Da daughter ions) was performed using optimized cone and collision energy voltages (23 V and 10 eV, respectively). Data were acquired using MassLynx 4.1 software (Waters). The same method was then used to fractionate the *ca.* 1.4 mg sample of the enantiomeric mixture of *iso*-petromyroxols (1). Two injections of 100 µL each were eluted and collected in *ca.* 40, 0.7 mL fractions. These were then individually analyzed by the above-described HPLC-MS/MS method. The appropriate fractions of each enantiomer were pooled and lyophilized, resulting in (+)-1 0.3 mg and (−)-1 0.5 mg of the faster and slower eluting enantiomers, respectively.

## 4. Conclusions

We have isolated and structurally characterized the *iso*-petromyroxols (**1**) and shown that they are diastereomeric with the dihydroxylated tetrahydrofuran-containing petromyroxols (**2**). The *iso*-petromyroxols represent a new addition to the molecular diversity of sea lamprey metabolites. The delineation of the absolute configuration of the *iso*-petromyroxols (**1**)—e.g., by Mosher ester analysis [18]—will be possible when larger amounts of enantiopure compounds become available.

## Supplementary Materials

Supplementary materials can be accessed at: http://www.mdpi.com/1420-3049/20/03/5215/s1.
