# Supplementary material for: iso-Petromyroxols: Novel Dihydroxylated Tetrahydrofuran Enantiomers from Sea Lamprey (Petromyzon marinus)
Source: Molecules. 2015 Mar 23;20(3):5215–22. doi: 10.3390/molecules20035215 (PMC6272722; doi:10.3390/molecules20035215)
Supplement: Supplementary file 1 [file molecules-20-05215-s001.pdf]

Supplementary Materials

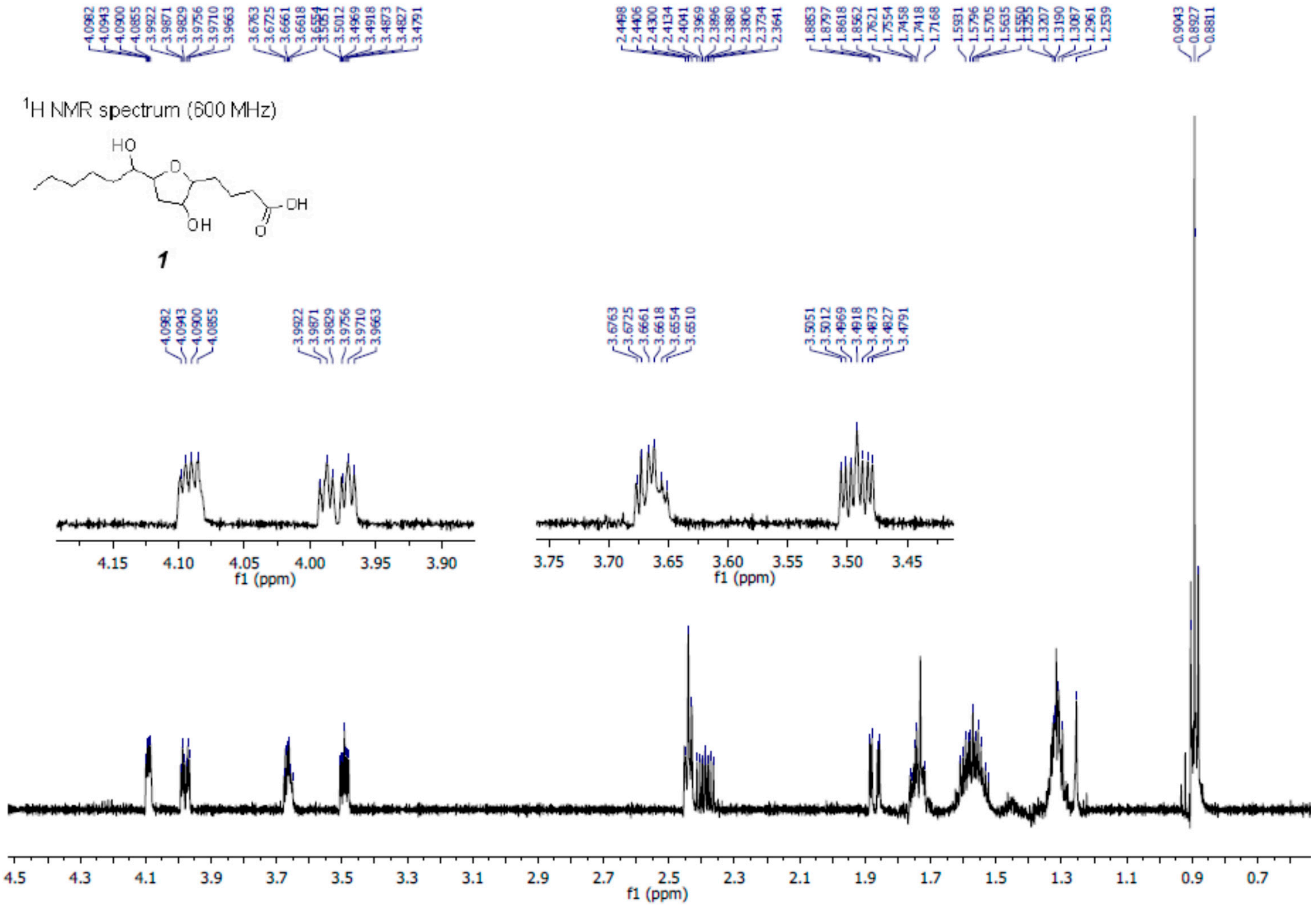

Figure S1. ^{1}H-NMR spectrum of *iso*-petromyroxol.

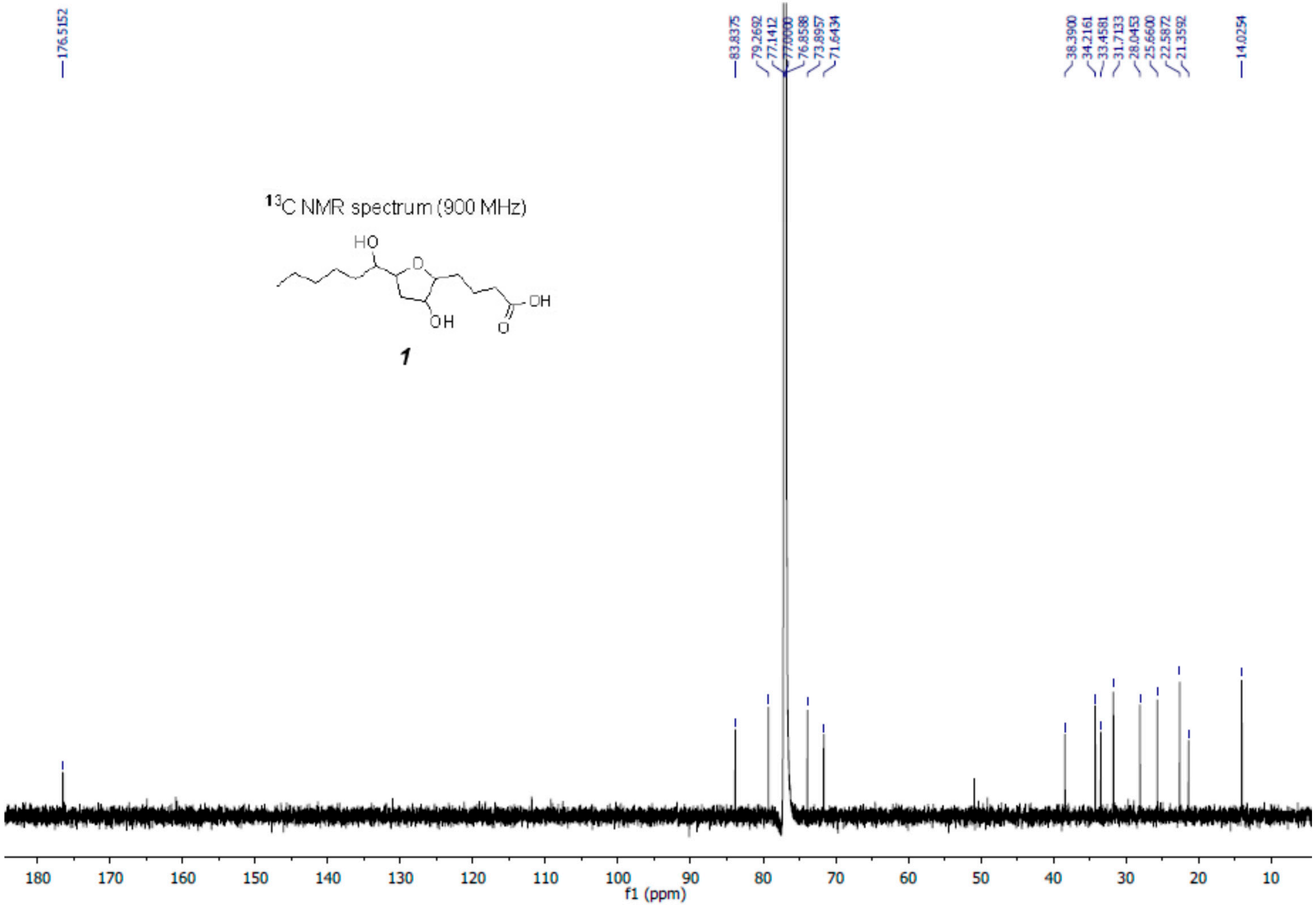

Figure S2. ^{13}C-NMR spectrum of *iso*-petromyroxol.

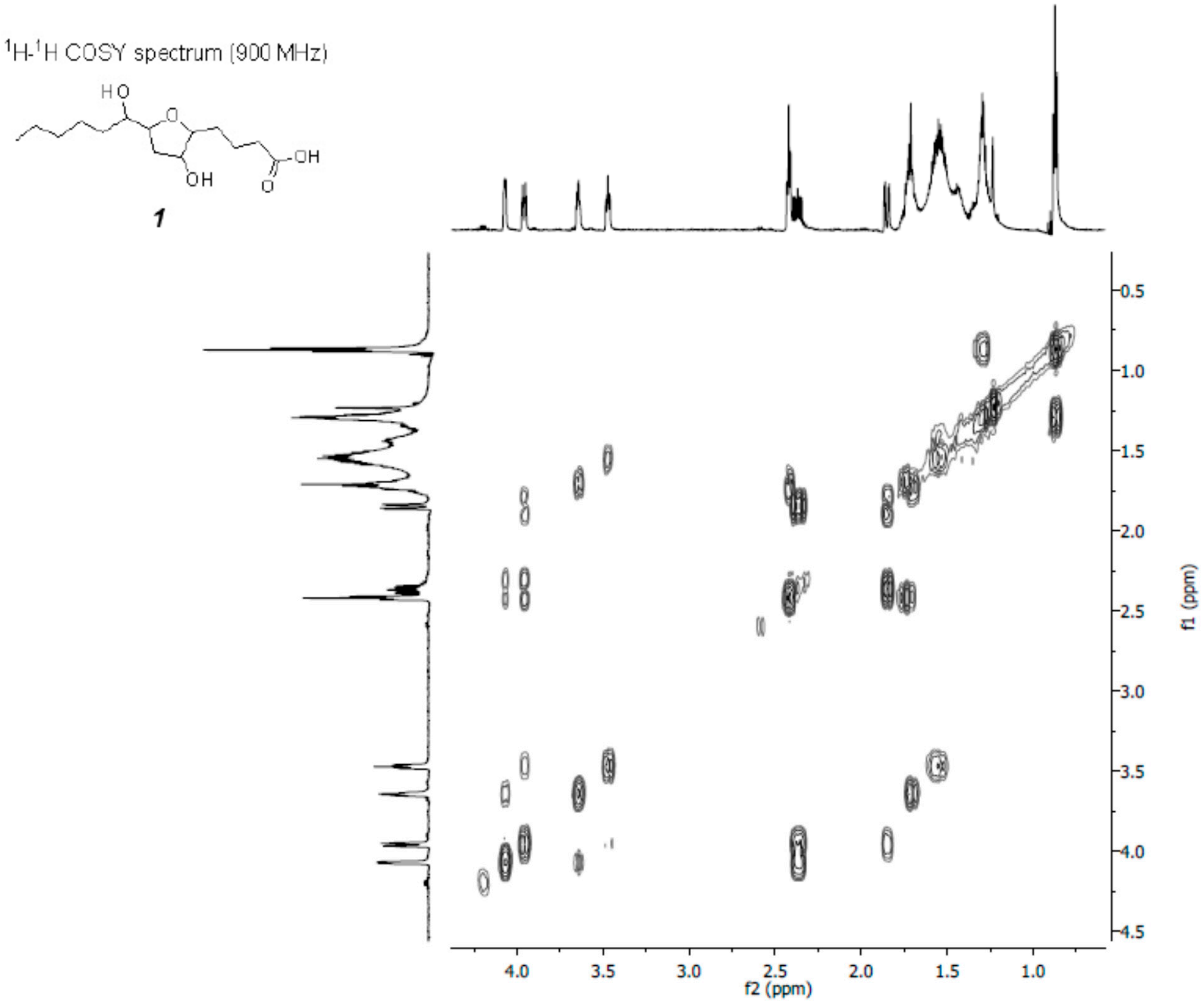

Figure S3. HSQC spectrum of *iso*-petromyroxol.

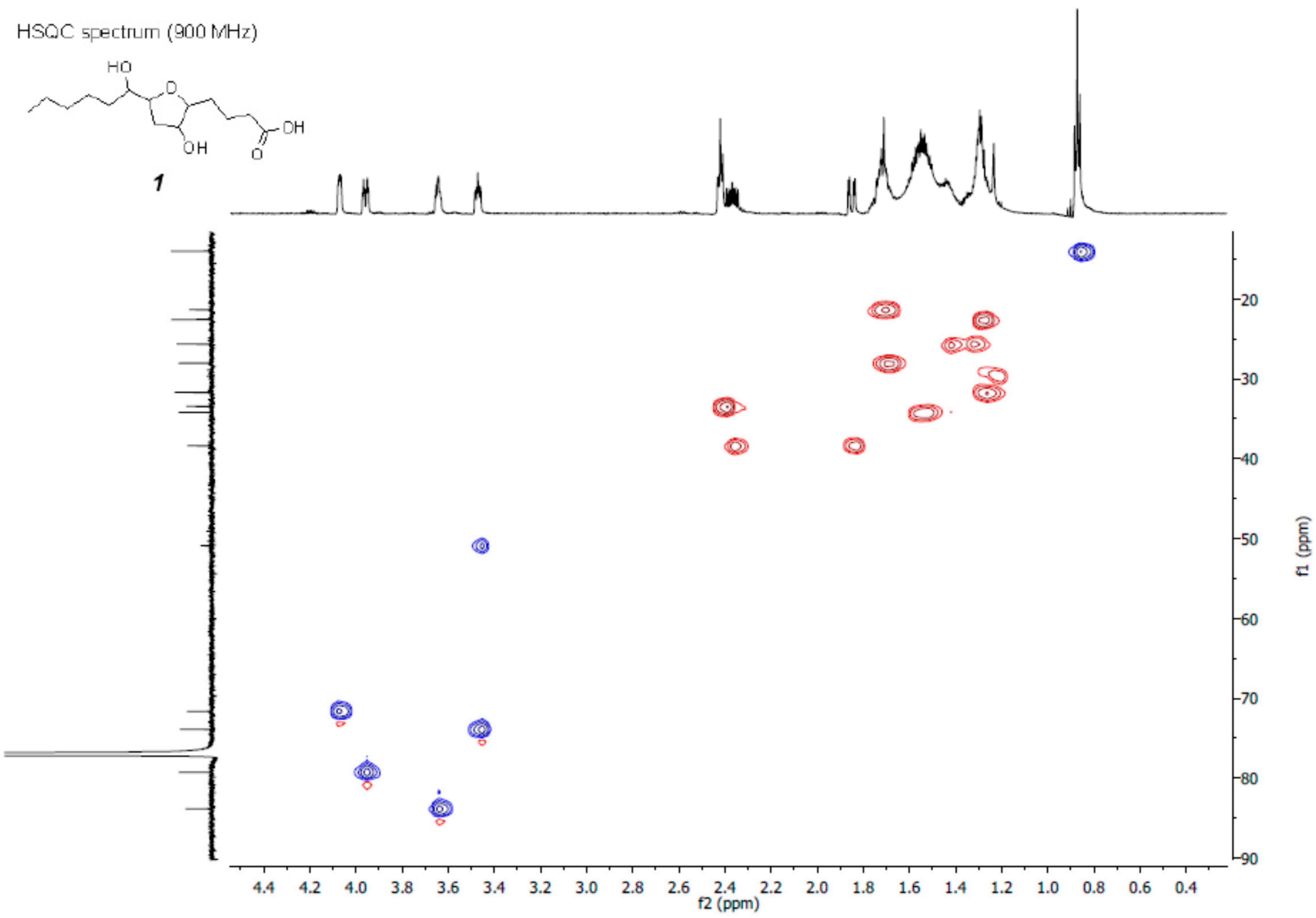

Figure S4. ^{1}H-^{1}H COSY spectrum of *iso*-petromyroxol.

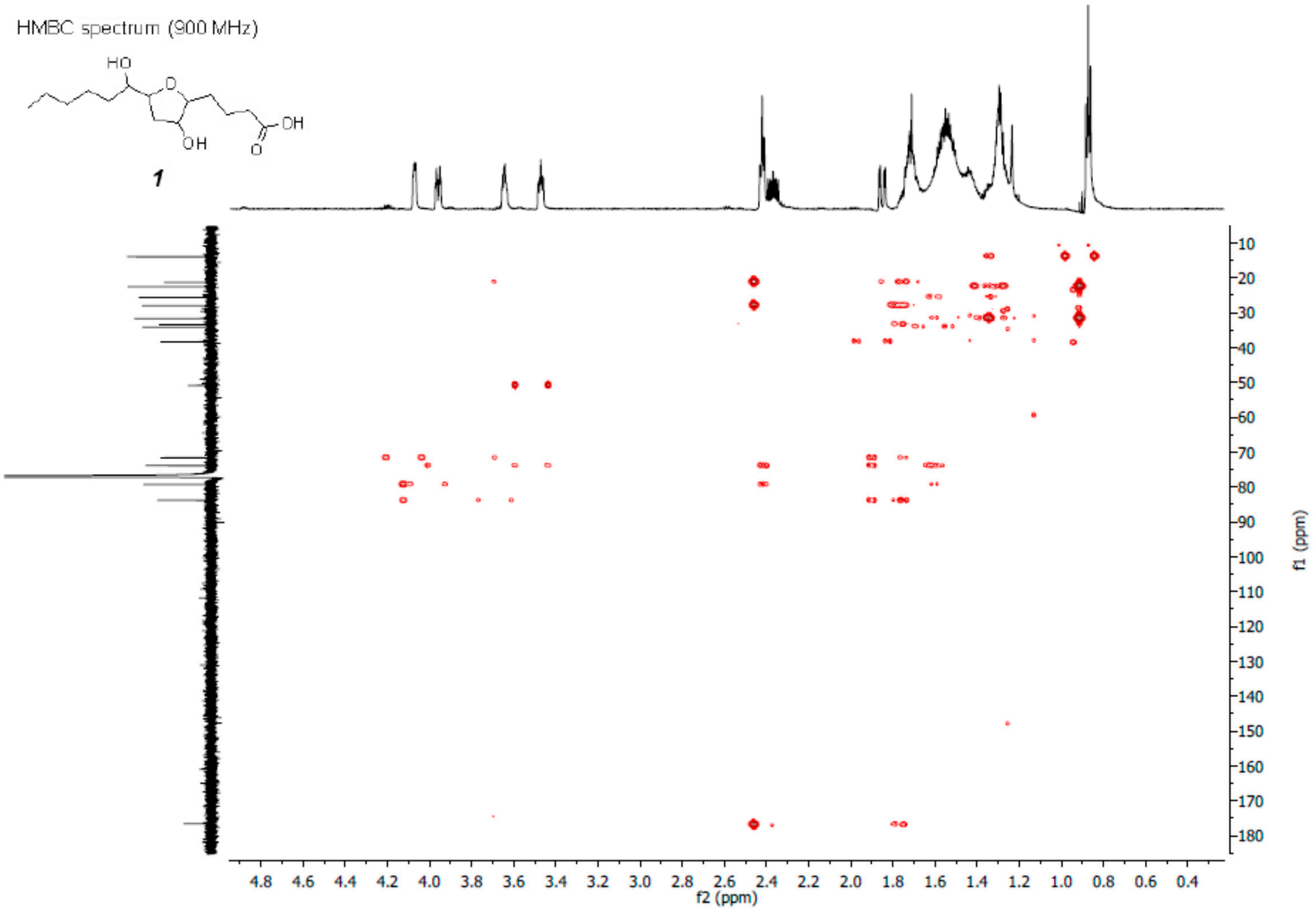

Figure S5. HMBC spectrum of *iso*-petromyroxol.

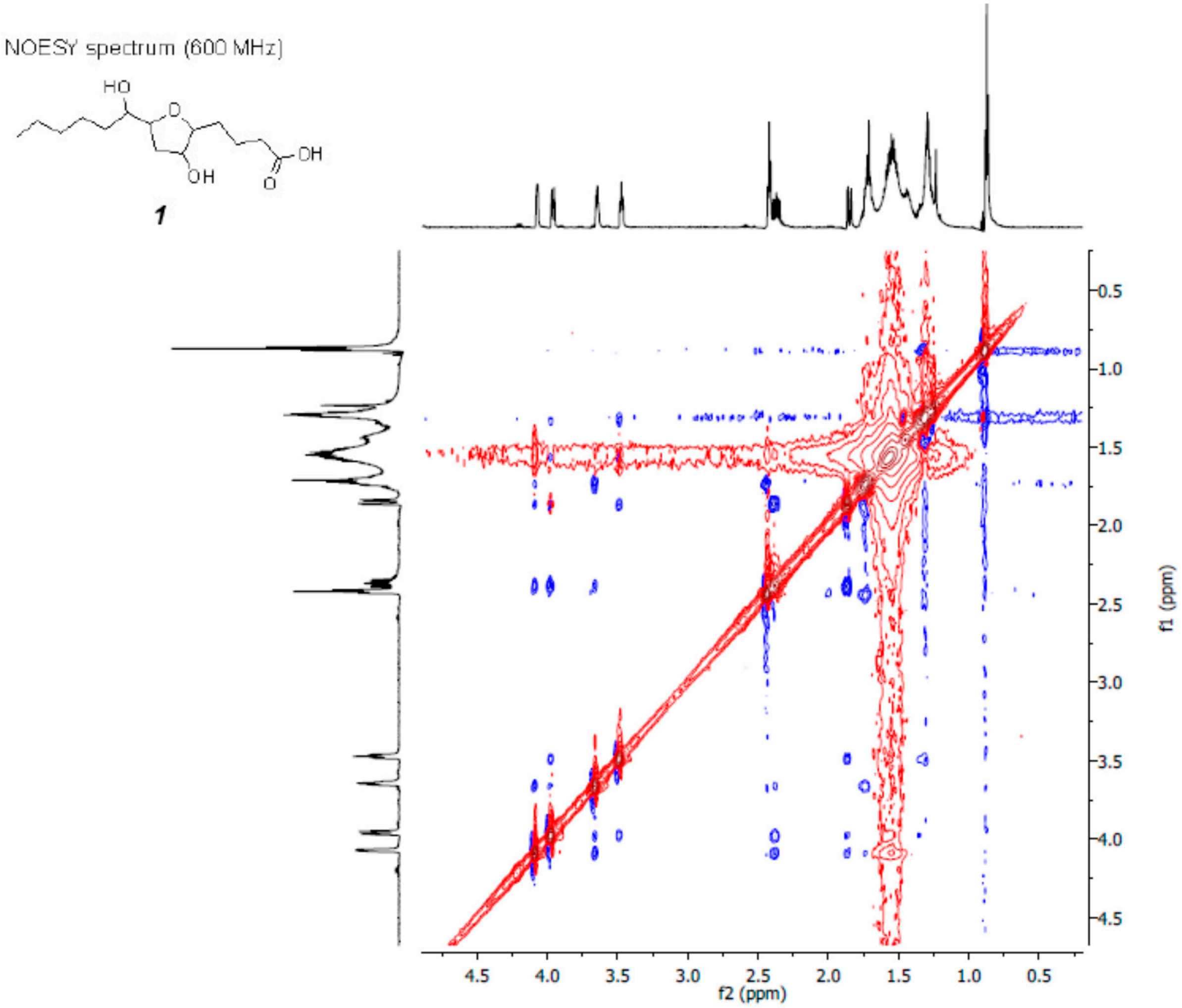

Figure S6. NOESY spectrum of *iso*-petromyroxol.
